# Effects of mental health status during adolescence on primary care costs in adulthood across three British cohorts

**DOI:** 10.1007/s00127-023-02507-y

**Published:** 2023-06-26

**Authors:** Derek King, Petra C. Gronholm, Martin Knapp, Mauricio S. Hoffmann, Eva-Maria Bonin, Nicola Brimblecombe, Rajendra Kadel, Barbara Maughan, Nick O’Shea, Marcus Richards, Ties Hoomans, Sara Evans-Lacko

**Affiliations:** 1 Care Policy and Evaluation Centre, London School of Economics and Political Science, London, UK; 2 Health Service and Population Research Department, Institute of Psychiatry, Psychology and Neuroscience, King’s College London, London, UK; 3 Department of Neuropsychiatry, Universidade Federal de Santa Maria, Avenida Roraima 1000, Building 26, Office 1446, Santa Maria, Brazil; 4 Universidade Federal do Rio Grande do Sul, Rua Ramiro Barcelos 2350, Porto Alegre, Brazil; 5 Public Health Wales, Policy and International Health Directorate, WHO CC on Investment for Health and Wellbeing, Cardiff, UK; 6 Social, Genetic and Developmental Psychiatry Centre, Institute of Psychiatry, Psychology and Neuroscience, King’s College London, London, UK; 7 Chief Economist, Centre for Mental Health, London, UK; 8 MRC Unit for Lifelong Health and Ageing at UCL, University College London, London, UK

**Keywords:** Mental health, Adolescence, Service costs, Primary care, General practice, Birth cohort

## Abstract

**Purpose:**

This study examines the association between mental health problems in adolescence and general practice (GP) costs during adulthood up to age 50 in the UK.

**Methods:**

We conducted secondary analyses of three British birth cohorts (individuals born in single weeks in 1946, 1958 and 1970). Data for the three cohorts were analysed separately. All respondents who participated in the cohort studies were included. Adolescent mental health status was assessed in each cohort using the Rutter scale (or, for one cohort, a forerunner of that scale) completed in interviews with parents and teachers when cohort members were aged around 16. Presence and severity of conduct and emotional problems were modelled as independent variables in two-part regression models in which the dependent variable was costs of GP services from data collection sweeps up to mid-adulthood. All analyses were adjusted for covariates (cognitive ability, mother's education, housing tenure, father's social class and childhood physical disability).

**Results:**

Adolescent conduct and emotional problems, particularly when coexisting, were associated with relatively high GP costs in adulthood up to age 50. Associations were generally stronger in females than males.

**Conclusion:**

Associations between adolescent mental health problems and annual GP cost were evident decades later, to age 50, suggesting that there could be significant future savings to healthcare budgets if rates of adolescent conduct and emotional problems could be reduced.

**Trial registration:**

Not applicable.

**Supplementary Information:**

The online version contains supplementary material available at 10.1007/s00127-023-02507-y.

## Background

There is convincing evidence that mental health problems in childhood and adolescence can impact society and public services. When children and young adults are persistently disruptive or anti-social, they generate immediate costs for treatment and support [1]. Mental health problems in childhood and adolescence are also linked with poorer health, socioeconomic and other social outcomes later in life [2–6]. Far less is known, however, about their long-term impacts on health services, including whether they affect use and costs of general practitioner (GP) services into adulthood.

General practice in the UK is usually the first point of contact for diagnosing, treating or onward referral to specialist care for health conditions. We focus on GP costs, given previous links between mood and impulse control disorders and chronic physical and mental health problems [7] and growing pressures on UK general practice due to rising demand and struggles to recruit and retain primary care staff [8, 9]. There is also evidence that prevalence of mental health problems, including depressive symptoms and anti-social behaviours, amongst adolescents are increasing, which could put further pressure on healthcare services [10–12]. Only a minority of people with mental health problems seek psychiatric treatment but may still seek GP support for other problems or problems which manifest as medically unexplained symptoms [13].

Using three historical British birth cohorts, we examine associations between adolescent mental health problems and costs of GP services used by those individuals up to age 50. We also extrapolate our estimates to project future primary care costs for the current population of UK adolescents.

## Methods

### Data sources

We use data from three British birth cohort studies: the MRC National Survey of Health and Development, the National Child Development Study and the 1970 British Cohort Study.

#### MRC national survey of health and development (NHSD)

A maternity survey of all births recorded in England, Scotland and Wales was initially conducted during one week in 1946 [14]. Of the 13,687 births, 5362 babies were selected for follow-up, comprising NSHD. Studies of the cohort have investigated numerous life-course predictors of health and wellbeing [15]. NSHD participants have been surveyed at 24 time points, most recently when aged 69. We use data collected from participants at ages 13 and 15 years to assess adolescent mental health. Data on GP visits were collected at ages 26, 31 and 43.

#### National child development study (NCDS)

The initial NCDS sample comprised 17,415 babies born in England, Scotland and Wales during one week in 1958 [16]. Data collection took place at various points in the life-course, with the sample augmented to include immigrants during childhood and adolescence. We use data collected at age 16 to assess adolescent mental health status and GP visits at ages 33, 42 and 50.

#### 1970 British cohort study (BCS70)

Designed to look at medical outcomes for 17,198 babies born in England, Scotland and Wales during a single week in 1970 [17], the BCS70 has since been expanded to collect data on educational, social and economic conditions. As in NCDS, subsequent child and adolescent waves incorporated new migrants to Britain born in the study sample birth week. Our analysis uses data collected at age 16 to assess mental health status and GP visits at ages 26, 30 and 38.

### Assessment of adolescent mental health status

Parents reported on cohort members in NCDS and BCS70 at age 16 using the Rutter scale measuring conduct problems, emotional problems and hyperactivity [18–20]. Teachers rated cohort members in the NSHD at ages 13 and 15 using a precursor of the Rutter scale measuring conduct and emotional problems [2]. Using established percentile cut points, we categorised conduct problems as severe (≥ 94%), mild/moderate (93–75%) or absent. We categorised emotional problems as severe (≥ 88%), mild/moderate (87–50%) or absent [2, 21–23].

The categories of severity for both conduct and emotional problems were dichotomised to presence (mild/moderate or severe) or absence (none). An overall severity variable was constructed, defined as the higher category on either conduct or emotional problems. That is, none if the adolescent had neither conduct nor emotional problems; mild or moderate if the adolescent was rated mild or moderate for either conduct or emotional problems; and severe if the adolescent was rated severe on either conduct or emotional problems.

### Individual-level GP costs in adulthood

To estimate costs of GP services for individual participants, we tracked their contacts with GPs during adulthood and multiplied these quantities by the unit cost of GP services. Use of GP services was assessed via questions relating to contacts with various health professionals and services at each data collection sweep included in our analyses (see Table 1). Using periodic surveys, members of the different cohorts were asked about their contacts with various health professionals and services. Question wording differed slightly across studies and sweeps. We considered any question about ‘seeing a doctor or GP’ for any reason, including after an accident, to reflect GP contact. We excluded any references to doctors working in specialty medical services or hospital visits and coded ‘don’t know’ responses as no contact.

**Table 1:** Distribution of demographic characteristics of 1948, 1958, 1970 cohort samples

Variable	NSHD (*n* = 5362)		NCDS (*n* = 18,558)		BCS (*n* = 19,023)	
	Assessment age	Percentage	Assessment age	Percentage	Assessment age	Percentage
Gender–female	Birth	47.5	Birth	48.3	Birth	48.0
Mother’s education–ompeted FT education at age 14/15 or above	Birth	14.7	Birth	17.6	Birth	34.1
Tenure–household home owned	2	23.8	6	42.2	10	61.5
Father’s social class	4		11		10	
I		5.4		4.5		4.9
II		16.9		19.5		23.4
III		42.6		52.0		51.1
IV		16.5		15.2		13.8
V		5.8		8.8		6.8
Disability^a^–Yes	13	5.9	16	7.7	16	1.2
General ability^b^–standardised: mean = 100; standard deviation = 15	11		11		10	
Type of mental health problems						
Conduct problems	15		16		16	
None		74.7		68.3		81.2
Mild/severe		25.3		31.7		18.9
Emotional problems	15		16		16	
None		50.0		49.6		51.7
Mild/severe		50.1		50.4		48.4
Overall severity^c^	15^d^		16^d^		16^d^	
None		34.5		37.2		45.0
Mild		45.9		41.0		33.5
Severe		19.6		21.8		21.5

^a^NCDS: parental interview form–NCDS Third follow-up, Question 66: taking into account the information you (interviewer) have obtained during the interview and any other relevant information, do you consider the child has any handicapping condition or disability?; BCS: parental interview form–question D7: does your teenager have an impairment, a disability or a handicap? (By ‘impairment’ we mean a physical or mental abnormality/illness. By ‘disability’ we mean difficulty in doing one or more mental or physical activities that average 16 year olds can do. By ‘handicap’, we mean a disability which interferes with the opportunities that others take for granted, e.g. problems with access/facilities in public buildings; not being considered for jobs he or she could manage if given a chance; other people are put off without even knowing what he or she is like). For NSHD, teachers were asked about the study subject if “school work adversely affected by any physical disability at 13 years”, with a yes/no answer

^b^NSHD: verbal and non-verbal ability devised by the National Foundation for Educational Research; NCDS: General Ability Scale in NCDS; BCS: British Ability Scale

^c^Highest level of severity of conduct and emotional problems

^d^Derived

All service use frequencies were standardised to one-year rates. It was necessary to estimate frequencies when these were not reported. For example, in the 1977 NSHD sweep, frequency of GP visits was not collected so we estimated frequencies from the nearest completed sweep (1989). For individuals where GP visit frequency was missing in 1989, we used the 1989 (gender-specific) median frequency from the subset of the sample who did report a GP visit and information on frequency of visits. More details on our methods of estimating frequency of GP visits are given elsewhere [24].

Year and age at which data were collected within each cohort is summarised in Supplementary Table 1.

We estimated annual GP costs and applied a £39.23 cost per GP visit at 2020 prices [25].

### Covariates

Previous literature informed our choice of covariates [2, 3, 26]. These were cognitive ability at age 11 (NSHD and NCDS) and age 10 (BCS70), mother’s level of education, housing tenure (family home owned vs rented), father’s social class (included as proxy for socioeconomic status of the family unit) and whether any physical disability was identified in childhood. Cognitive ability was assessed in NSHD using tests of verbal and non-verbal ability tests devised by the National Foundation for Educational Research [27], the General Ability Test [28] in NCDS and the British Ability Scales [29] in BCS70. All scores were transformed into mean IQ equivalents of 100 and standard deviations of 15 [30]. Mother’s education was dichotomised as 0 if ceased full-time education before age 16 (age 14/15 in NSHD) and 1 if full-time education was continued to age 16 or beyond (age 14/15 or beyond in NSHD). For NCDS and BCS70, social class was based on father’s occupation. If these data were not available for the child’s father, their mother’s occupation was substituted. For NSHD, a derived variable of father’s social class was included in the dataset.

### Statistical analyses

The three birth cohorts were analysed separately. We established distributions of socio-demographics for each sample. (For bivariate comparisons between socio-demographic characteristics and adolescent mental health status for each of the cohorts, see Supplementary Tables 2, 3 and 4). We then calculated the percentage of missing values in these data and GP visits at each follow-up. Data could be missing due to non-response on a particular question or attrition over time.

We assumed that cohort data were missing at random—implying that systematic differences between missing values and observed ones can be explained by observed data (and not on unobserved variables) [31]. On the basis of this (and previous work on the datasets [32, 33]), we choose to impute missing data using multiple imputation with chained equations.

Imputation samples included all participants with data on adolescent mental health measures. We incorporated into the imputations indices of mental health problems, covariates and health care costs at each follow-up visit. To improve model efficiency, we added auxiliary variables that earlier were found to be highly correlated with explanatory variables and variables that explain the mechanism leading to missing data [26]. Those auxiliary variables included the number of previous pregnancies of the mother (except NSHD); whether the child was breastfed; whether the mother lived in South-East England; her age at the child’s birth; and whether she was married at the time of the child’s birth. Ten datasets with imputed values were created.

We analysed associations between adolescent mental health and GP costs using two-part models [34, 35], with data clustered by cohort participant to reflect within-participant correlation of GP costs over time. The first-part model used logistic regression to estimate associations between use of GP services (dependent variable coded ‘yes/no’) and presence and severity of adolescent mental health problems, adjusting for the covariates. We also added a dummy variable for ‘year’ in each model.

The second-part model assessed associations between presence and severity of adolescent mental health problems and annual GP costs amongst those who had visited their GP, again adjusting for covariates and year. We used a generalised linear gamma model where the dependent variable, GP costs, was first log-transformed. Separate models were run by gender, as the prevalence of severe conduct and emotional problems differed by gender across the three cohorts [24].

Predicted mean GP costs for combinations of type, severity and comorbidity of mental health problems according to gender were estimated using two-part multilevel gamma regression models [36]. The two-part multilevel model combines a multilevel logistic regression and a multilevel gamma regression. Predicted values from the two parts are multiplied to estimate the predicted GP costs for a given year between early and mid-adulthood. These predicted costs, and their 95% confidence intervals, were graphed to illustrate the estimates of GP costs across each combination of effects of mental health problems. Models are again two-part, but without the multilevel (across sweeps) component: a logistic regression model of use (yes/no) of GP services and a generalised linear model of GP costs amongst those who saw their GP using a log-link function and a gamma distribution with adolescent mental health problems as independent variables and adjusted for covariates identified above and the year for which GP costs were estimated. To facilitate comparisons, we estimated absolute monetary values (by adolescent mental health type and severity) and relative differences (in percentages).

### Expected population-level GP costs adulthood associated with adolescent mental health problems

To estimate impact of adolescent mental health problems on GP costs, we first estimated total number of adolescents (males and females) in the different mental health status categories (ranging from experiencing no conduct or emotional problems to severe mental health problems) in 2020 using established category percentile cut points [21–23] and Office of National Statistics data [37].

In mid-2020, there were 380,328 males and 360,365 females aged 16 years [37]. Based on the percentile cuts described above, if 6% of adolescents are assumed to have severe conduct problems and 12% severe emotional problems, there would have been, nationally in 2020 approximately 22,820 adolescent boys and 21,620 adolescent girls with severe conduct problems, and approximately 45,640 adolescent boys and 43,240 adolescent girls with severe emotional problems. The numbers with comorbid conduct and emotional problems were 16,350 adolescent boys and 16,680 adolescent girls.

We multiplied these estimated totals by the estimated annual GP costs in adulthood for each combination of gender and type of mental health problem as estimated by our individual-level impact estimation models. This estimates future annual population-level excess adulthood GP costs incurred by the NHS in the UK due to the prevalence of conduct or emotional problems in adolescents in 2019, if treatment and support for this cohort of adolescents, now and in the future, remains unchanged. We discounted future costs at a 3% rate.

Analyses were conducted in STATA version 15 [38].

## Results

Table 1 summarises the characteristics of members of the three cohorts. A minority of NSHD and NCDS grew up in owner-occupied housing, compared to the majority of BCS70. The distribution of social class based on father’s occupation showed a small trend in upward mobility across cohorts. Mental health status also seems to differ according to demographics (see Supplementary Tables 2, 3 and 4). This provides additional justification for controlling for these variables when estimating associations between adolescent mental health and adulthood GP costs.

### Individual-level impact

#### NSHD (1946 cohort)

**Fig. 1:**
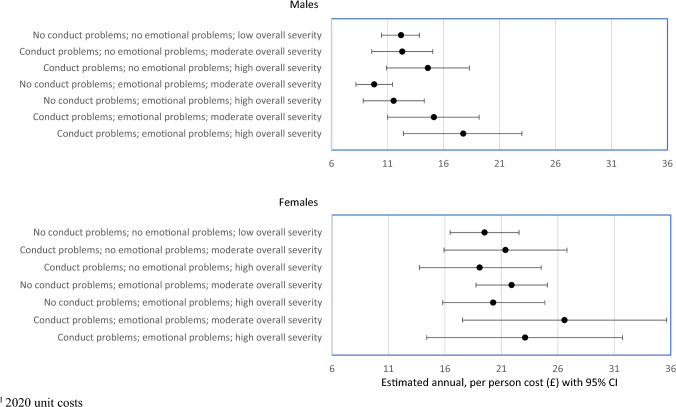
MRC National Survey of Health and Development: 1946 cohort–estimated annual GP costs^1^ by mental health status at 13 and 15 years of age

Figure 1 shows estimated mean annual GP costs during adulthood for men and women in NSHD, by mental health status. Mean costs were £12.24 (95% CI £10.52 to £13.96) for men without conduct or emotional problems during adolescence. Having had conduct problems (but not emotional problems) or emotional problems (but not conduct problems) in adolescence was not associated with significantly increased costs (£12.30, 95% CI £9.58 to £14.46 and £9.77, 95% CI £8.12 to £11.45, respectively). Having both condition was associated with higher costs (£15.16, 95% CI £11.09 to £19.24). When one or both conditions was severe, mean annual costs rose to £17.81 (95% CI £12.48 to £23.14). Estimated annual GP costs for women who did not experience conduct or emotional problems in adolescents were £19.51 (95% CI £16.47 to £22.55), approximately 60% higher than for corresponding men in the NSHD cohort. Estimated annual GP costs for women were higher costs when both conditions coexisted at a moderate level (£26.60, 95% CI £17.55 to £35.64).

#### NCDS (1958 cohort)

**Fig. 2:**
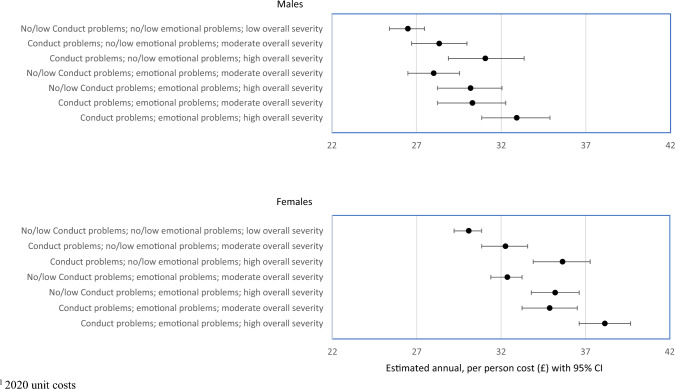
National Child Development Study: 1958 cohort–estimated annual GP costs^1^ by mental health status at 16 years of age

Figure 2 shows estimated mean annual GP costs incurred during adulthood by men and women in NCDS, respectively.

There is a statistically significant association between adolescent mental health and adulthood GP costs for men, with those experiencing severe and concurrent conduct and emotional problems in adolescence generating mean annual GP costs of £32.92 (95% CI £30.97 to £34.96) in adulthood and those without conduct or emotional problems in adolescence £26.54 (95% CI £25.53 to £27.56). Men also generated higher GP costs in adulthood if they experienced severe conduct problems, severe emotional problems or concurrent moderate conduct and emotional problems during adolescence.

Similar patterns were found for women: estimated mean annual GP costs in adulthood were £30.13 (95% CI £29.31 to £30.93) for those without conduct and emotional problems in adolescence compared to £38.16 (95% CI £36.62 to £39.71) when both were present and one or both was severe. Note that, as in NSHD, born 12 years earlier, mean annual GP costs in adulthood were higher for women than for men when comparing those not experiencing conduct or emotional problems in adolescence (£30.13 vs. £26.54 with non-overlapping confidence intervals).

#### BCS70 (1970 cohort)

**Fig. 3:**
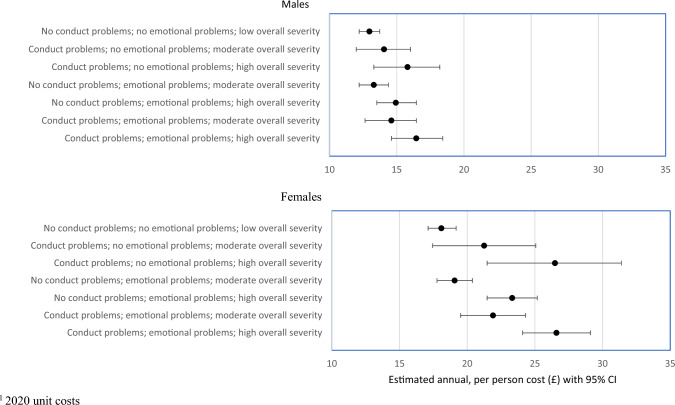
British Cohort Study: 1970 cohort–estimated annual GP costs^1^ by mental health status at 16 years of age

Figure 3 shows the equivalent findings for men and women in BCS70, born 12 years after the NCDS sample and 24 years after the NSHD sample. Amongst men, estimated annual GP costs in adulthood were significantly higher for those with severe conduct problems in adolescence (£15.85, 95% CI £13.36 to £18.33) compared to the group where neither adolescent conduct nor emotional problems were present (£12.95, 95% CI £12.15 to £13.74). Having had emotional problems in adolescence was not significantly associated with higher GP costs in adulthood (£13.29, 95% CI £12.23 to £14.34), though if either or both conduct and emotional problems were *severe*, costs were significantly higher (£16.43, 95% CI £14.49 to £18.37).

We also found an association for women in this cohort. Mean annual GP costs during adulthood are estimated to be £18.04 (95% CI £17.00 to £19.08) for those without conduct and emotional problems. If severe conduct problems were present, mean costs were estimated as £26.52 per year (95% CI £21.60 to £31.45); if severe emotional problems were present, mean costs were estimated as £23.34 per year (95% CI 21.53 to £25.15), which is statistically significantly greater than for women with no conduct and emotional problems during adolescence.

#### Patterns across cohorts

Some consistent patterns were observed when examining the results from the three cohorts. First, baseline costs for females (with no problems) tended to be higher than for males. Second, in almost all scenarios (16 out of 18), the percentage increase in costs attributable to adolescent mental health problems were greater for females than for males, though in some cases the percentage increases were relatively small (see Table 2 and Figs. 1, 2 and 3). Average cost appears to be greater for females, though also with wider confidence intervals. Third, severity and comorbidity make a difference. Presence of a single morbidity did not always increase costs, but for those with both conduct and emotional problems, and where at least one represented high severity, costs increased by amounts ranging from 18 to 47%.

**Table 2:** Predicted base case annual GP costs (£) in adulthood and percentage change associated with presence and severity of mental health problems in adolescence; estimated using a two-part multilevel model

	NSHD cohort models		NCDS cohort models		BCS cohort models	
	Male	Female	Male	Female	Male	Female
Predicted base case annual GP costs (£)						
No conduct problems; no emotional problems; low overall severity	19.51	12.21	30.08	26.49	18.09	12.97
Percentage change in cost						
Conduct problems; no emotional problems; high overall severity	− 2.2%	19.6%	18.5%	17.3%	46.4%	21.8%
No conduct problems; emotional problems; moderate overall severity	12.3%	− 19.6%	7.6%	5.8%	5.4%	2.5%
No conduct problems; emotional problems; high overall severity	3.9%	− 5.4%	17.0%	14.0%	28.9%	15.1%
Conduct problems; emotional problems; moderate overall severity	36.3%	24.1%	15.9%	14.4%	21.1%	12.6%
Conduct problems; emotional problems; high overall severity	18.4%	45.5%	26.8%	24.3%	47.0%	26.9%

### Future cost implications at a national level

Using results from our NSHD models, national annual GP costs in adulthood would be £10,721,617 if no conduct or emotional problems were present in adolescence. Presence of severe conduct problems (and no emotional problems) increases annual GP costs in adulthood by £23,997; severe emotional problems (and no conduct problems) by £3045. Presence of comorbid conduct and emotional problems increases annual GP costs in adulthood by £89,684. Nationally, these costs represent an additional 612 and 78 GP visits annually, respectively, attributable to adolescent conduct and emotional problems, and an additional 2286 GP visits attributable to comorbid conduct and emotional problems.

Similar calculations can be made using the NCDS and BCS70 models. From our NCDS findings, annual GP costs if no conduct or emotional problems were present in adolescence were estimated to be £19,221,475 nationally. Severe conduct problems (and no emotional problems) increase costs by £134,282 and severe emotional problems (and no conduct problems) increase costs by £307,585. Comorbid conduct and emotional problems increase estimated GP costs by £233,313. This would represent an additional 3423 and 7841 GP visits nationally per year attributable to adolescent conduct and emotional problems, respectively, and an additional 5947 GP visits attributable to comorbid conduct and emotional problems.

From our BCS70 models, estimated total annual cost of GP visits across adulthood if no adolescents experienced conduct or emotional problems is £10,482,337. Severe conduct problems in adolescence (and no emotional problems) increase costs by £124,830, and severe emotional problems by £246,890. Comorbid conduct and emotional problems in adolescents are associated with an increase in adulthood GP costs of £254,900. These represent 3182 and 6293 additional GP visits nationally per year attributable to adolescent conduct problems and emotional problems respectively, and 6,498 additional visits attributable to comorbid conduct and emotional problems.

## Discussion

By attaching costs to reports of GP visits, we found that adolescent conduct and emotional problems, particularly when coexisting, are associated with higher costs to the NHS into adulthood. The associations were stronger in females than males in the two later cohorts. Pressure on primary care services could be eased if conduct and emotional problems were prevented or successfully managed in adolescence. This is particularly important given evidence that rates of adolescent mental health problems are growing [11, 12] and associated impacts are worsening [39].

### Strengths and limitations

We used three large, nationally representative datasets and new computations to calculate and compare costs of primary care contacts in adulthood across subgroups differentiated by their mental health experiences in adolescence. Comparisons between the cohorts should be made cautiously because of differences in question scope and wording. We identified some of the challenges of cross-study comparisons and suggested methods for dealing with these based on published guidance [40]. For example, we established percentage cost differences between groups and genders to facilitate inter-cohort comparisons. There were also changes in question scope and wording at different data collection points within cohorts; for example, in NSHD at age 26, cohort members were asked about consultations with GPs due to accidents only and thus underestimated total GP costs. At age 31, frequency of GP consultations was not collected in NSHD and had to be imputed from the next available sweep. GP service use was based on self-report data (usually, recall of number of consultations in the previous year) and may under-estimate the true figure, although previous evidence is not consistent [41, 42].

Timing of assessment of adolescent mental health problems captured a limited window of exposure. Emotional problems may emerge later in adolescence than the age (around 16) at which assessments were made [43, 44]. More cohort members would have been identified with conduct and/or emotional problems if data collection had covered a wider age range in adolescence.

By analysing the different cohorts separately, we did consider potential cohort effects and were able to identify and describe patterns in adolescent mental health, GP costs and associations over time that were both cohort-specific and consistent between cohorts. There may also be differences across cohorts and sweeps in rates of adolescent mental health problems or access and availability of GP services that we haven’t accounted for. It is unlikely, however, that these have changed over time differentially between those with and without mental health problems. What is clear, however, is that differences in GP costs between groups of adolescents defined by their mental health status are found consistently across all three cohorts.

We focussed on costs associated with GP services, an area which has received limited research attention. We did not include costs for other health service contacts. However, GPs are often the first point of contact for people with mental health problems, both for mental health support and to address physical health needs, and in Britain are the gateways to specialist care, hospital services and diagnostic tests [45]: their actions are consequently often pivotal for further service utilisation and costs. Nonetheless, differences in GP costs reflect only a proportion of the potential difference in health service use and costs attributable to poor adolescent mental health. For example, for those children and adolescents with a psychiatric condition in Britain, average cost per user of specialty mental health services was about four times greater than for primary care services; however, more than twice as many young people with a psychiatric condition saw a GP compared with a psychologist or psychiatrist [46]. Even for GP costs only, however, our estimates will almost certainly be conservative by comparisons with today’s costs as we know current mental health problem prevalence is higher today than for adolescent cohort participants [47].

### Comparisons with previous research

Our findings support and expand those from the few previous studies addressing long-term economic impacts of child and adolescent mental health on GP services specifically. The first study to investigate longer-term economic impacts of childhood mental illness, conducted in inner London, found that, compared to those with no disorder, individuals with conduct disorder incurred significantly higher GP costs to age 28 [48]. They also incurred higher inpatient costs. Another London study examined long-term (21-year follow-up) economic impacts associated with child and adolescent depression, finding that that comorbidity with conduct disorders was associated with persistently higher total healthcare costs, although there was no significant difference for GP costs specifically [49].

Primary care arrangements are country-specific, which potentially influences service cost patterns, but evidence from outside the UK also shows that child mental health conditions influence GP costs. Findings for the Dunedin cohort in New Zealand, which distinguished different trajectories of conduct problem subtypes, suggest that children with conduct problems that persisted into adulthood reported significantly more emergency department visits and prescription fills than those without persistent conduct problems [50]. Our study, by comparing results across cohorts, conducting separate analysis by gender, and by analysing the additional effect of emotional problems, provides further support for understanding the impacts of conduct problems in adolescence and identifying subgroups with higher costs. By assessing conduct problems in adolescence, we have likely picked up both life-course persistent and adolescent-limited conduct problems [51], but differences in GP costs may have been more pronounced had we been able to distinguish between those whose onset of conduct problems began in childhood and persisted into adulthood compared to those whose conduct problems did not persist into adulthood.

### GP service cost implications according to gender

In all three cohorts, females had higher primary care costs associated with adolescent mental health problems than did males, with the additional finding that adolescent mental health conditions were a greater driver of GP costs in adulthood for females in 16 of the 18 scenarios. This is consistent with other research which found that female adults tend to use more primary care health services overall, and in relation to mental health problems, even when controlling for greater prevalence of underlying morbidity [52]. Interestingly, the gender differential seems to be greater for primary versus specialty care when seeking support for mental health problems [53]. Higher rates of primary care or GP visits may indicate greater willingness to seek help early on amongst females compared with males. There is some research suggesting that males underutilise preventive care [54], which could lead to greater costs later, such as for hospital and emergency care services [55]. Thus, lower GP costs amongst males may reflect underutilisation of GP services by males, which could have knock-on impacts in other areas such as employment or productivity [56].

## Conclusion

Estimating the additional adulthood costs linked to adolescent mental health problems shows some of the savings to public health budgets that could be realised if adolescent conduct and emotional problems could be prevented or treated early. Of course, there are larger economic impacts beyond GP service utilisation, including secondary healthcare costs, lost productivity, crime and criminal justice contacts, welfare benefit payments and social immobility [3, 7, 57]. Nevertheless, our focus on primary care services points to, and quantifies, the added pressures on what are already very stretched services that stem from mental health problems earlier in life. Investing in actions to prevent or interventions to alleviate conduct and emotional problems in adolescence would help to reduce primary care and other impacts in adulthood.

### Supplementary Information

Below is the link to the electronic supplementary material.

Supplementary file1 (DOCX 28 kb)

## Data Availability

This publication is based on multiple datasets which are openly available from the UK Data Service.

## References

[1] Romeo R, Knapp M, Scott S (2006). Economic cost of severe antisocial behaviour in children – and who pays it. Br J Psychiatry.

[2] Richards M, Abbott R, Collis G, Hackett P, Hotopf M, Kuh D, Jones P (2009). Childhood mental health and life chances in post-war Britain: insights from three national birth cohort studies.

[3] Goodman A, Joyce R, Smith JP (2011). The long shadow cast by childhood physical and mental problems on adult life. Proc Natl Acad Sci.

[4] Knapp M, King D, Healey A, Thomas C (2011). Economic outcomes in adulthood and their associations with antisocial conduct, attention deficit and anxiety problems in childhood. J Ment Health Policy Econ.

[5] Beecham J (2014). Annual research review: Child and adolescent mental health interventions: a review of progress in economic studies across different disorders. J Child Psychol Psychiatry.

[6] Rodwell L, Romaniuk H, Nilsen W, Carlin JB, Lee KJ, Patton GC (2018). Adolescent mental health and behavioural predictors of being NEET: a prospective study of young adults not in employment, education, or training. Psychol Med.

[7] Scott KM, Lim C, Al-Hamzawi A, Alonso J, Bruffaerts R, Caldas-de-Almeida JM (2016). Association of mental disorders with subsequent chronic physical conditions. JAMA Psychiat.

[8] Baird B, Charles A, Honeyman M, Maguire D, Das P (2016) Understanding pressures in general practice. The King’s Fund, London. http://www.kingsfund.org.uk/publicatioins/general-practice. Accessed 10 Aug 2022

[9] British Medical Association (2022) Pressures in general practice data analysis. British Medical Association, London. https://www.bma.org.uk/advice-and-support/nhs-delivery-and-workforce/pressures/pressures-in-general-practice-data-analysis. Accessed 10 Aug 2022

[10] Patalay P, Gage SH (2019). Changes in millennial adolescent mental health and health-related behaviours over 10 years: a population cohort comparison study. Int J Epidemiol.

[11] Sweeting H, Young R, West P (2009). GHQ increases among Scottish 15 year olds 1987–2006. Soc Psychiatry Psychiatr Epidemiol.

[12] Collishaw S, Maughan B, Natarajan L, Pickles A (2010). Trends in adolescent emotional problems in England: a comparison of two national cohorts twenty years apart. J Child Psychol Psychiatry.

[13] Evans-Lacko S, Aguilar-Gaxiola S, Al-Hamzawi A, Alonso J, Benjet C, Bruffaerts R (2017). Socio-economic variations in the mental health treatment gap for people with anxiety, mood, and substance use disorders: results from the WHO World Mental Health (WMH) surveys. Psychol Med.

[14] Wadsworth MEJ (1969). The national survey of health and development. Health Visit J.

[15] Khanolkar AR, Chaturvedi N, Kuan V, Davis D, Hughes A, Richards M, Bann D, Patalay P (2021). Socioeconomic inequalities in prevalence and development of multimorbidity across adulthood: a longitudinal analysis of the MRC 1946 National Survey of Health and Development in the UK. PloS Med.

[16] Power C, Elliott J (2006). Cohort profile: 1958 British birth cohort (National Child Development Study). Int J Epidemiol.

[17] Elliott J, Shepherd P (2006). Cohort profile: 1970 British Birth Cohort (BCS70). Int J Epidemiol.

[18] Rutter M (1967). A children’s behaviour questionnaire for completion by teachers: preliminary findings. J Child Psychol Psychiatry.

[19] Rutter M, Tizard J, Whitmore K (1970). Education, health and behaviour.

[20] Elander J, Rutter M (1996). An update on the status of the Rutter Parents’ and Teachers’ Scales. Child Psychol Psychiatry Rev.

[21] Ghodsian M, Fogelman K, Lambert L, Tibbenham A (1980). Changes in behaviour ratings of a national sample of children. Br J Soc Clin Psychol.

[22] Rodgers B (1990). Behaviour and personality in childhood as predictors of adult psychiatric disorder. J Child Psychol Psychiatry.

[23] Colman I, Murray J, Abbott RA, Maughan B, Kuh D, Croudace TJ, Jones PB (2009). Outcomes of conduct problems in adolescence: 40 year follow-up of national cohort. BMJ.

[24] Gronholm PC, Knapp M, Brimblecombe N, Maughan B, Richards M, Bonin E, Hoffmann MS, Kadel R, King D, Hoomans T, O’Shea N, Evans-Lacko S (2022). Estimating health service costs in early- and mid-adulthood associated with adolescent mental health problems: data from three British birth cohorts. J Ment Health Policy Econ.

[25] Jones K, Burns A (2021) Unit Costs of Health and Social Care 2021. Personal Social Services Research Unit, Canterbury

[26] Mostafa T, Narayanan M, Pongiglione B, Dodgeon B, Goodman A, Silverwood RJ, Ploubidis GB (2020). Missing at random assumption made more plausible: evidence from the 1958 British birth cohort. J Clin Epidemiol.

[27] Richards M, Stephen A, Mishra G (2010). Health returns to cognitive capital in the British 1946 birth cohort. Longitud Life Course Stud.

[28] Douglas JWB (1964). The home and the school: a study of ability and attainment in the primary school.

[29] Elliott CD, Murray DJ, Pearson LS (1979) British Ability Scales. National Foundation for Educational Research, Slough

[30] Gale CR, Hatch SL, Batty GC, Deary IJ (2009). Intelligence in childhood and risk of psychological distress in adulthood: the 1958 national child development survey and the 1970 British cohort study. Intelligence.

[31] Little RJA, Rubin DB (2002). Statistical analysis with missing data.

[32] McElroy D, Richards M, Fitzsimons E, Conti G, Ploubidis GB, Sullivan A, Moulton V (2021). Influence of childhood socioeconomic position and ability on mid-life cognitive function: evidence from three British birth cohorts. J Epidemiol Community Health.

[33] White J, Fluharty M, de Groot R, Bell S, Batty GD (2022). Mortality among rough sleepers, squatters, residents of homeless shelters or hotels and sofa-surfers: a pooled analysis of UK birth cohorts. Int J Epidemiol.

[34] Mullahy J (1998). Much ado about two: reconsidering retransformation and the two-part model in health economics. J Health Econ.

[35] Deb P, Norton EC (2018). Modeling health care expenditures and use. Annu Rev Public Health.

[36] Baldwin SA, Fellingham GW, Baldwin AS (2016). Statistical models for multilevel skewed physical activity data in health research and behavioral medicine. Health Psychol.

[37] Office of National Statistics (2020) Mid-year population estimates, UK, June 2020. Office of National Statistics, London. http://www.ons.gov.uk. Accessed 12 May 2022

[38] StataCorp, (2017). Stata 15 base reference manual.

[39] Sellers R, Warne N, Pickles A, Maughan B, Thapar A, Collishaw S (2019). Cross-cohort change in adolescent outcomes for children with mental health problems. J Child Psychol Psychiatr.

[40] Bann D, Wright L, Goisis A, Hardy R, Johnson W, Maddock J, McElroy E, Moulton V, Patalay P, Scholes S, Silverwood RJ, Ploubidis GB, O’Neill D (2022). Investigating change across time in prevalence or association: the challenges of cross-study comparative research and possible solutions. Discov Soc Sci Health.

[41] Short ME, Goetzel RZ, Pei X, Tabrizi MJ, Ozminkowski RJ, Gibson TB, Dejoy DM, Wilson MG (2009). How accurate are self-reports? Analysis of self-reported health care utilization and absence when compared with administrative data. J Occup Environ Med.

[42] Brusco NK, Watts JJ (2015). Empirical evidence of recall bias for primary health care visits. BMC Health Serv Res.

[43] Rutter M, Kim-Cohen J, Maughan B (2006). Continuities and discontinuities in psychopathology between childhood and adult life. J Child Psychol Psychiatry.

[44] Colman I, Ploubidis GB, Wadsworth MEJ, Jones PB, Croudace TJ (2007). A longitudinal typology of symptoms of depression and anxiety over the life course. Biol Psychiatry.

[45] Greenfield G, Foley K, Majeed A (2016). Rethinking primary care’s gatekeeper role. BMJ.

[46] Snell T, Knapp M, Healey A, Guglani S, Evans-Lacko S, Fernandez JL, Meltzer H, Ford T (2013). Economic impact of childhood psychiatric disorder on public sector services in Britain: estimates from national survey data. J Child Psychol Psychiatry.

[47] Cybulski L, Ashcroft DM, Carr MJ, Garg S, Chew-Graham CA, Kapur N, Webb RT (2021). Temporal trends in annual incidence rates for psychiatric disorders and self-harm among children and adolescents in the UK, 2003–2018. BMC Psychiatry.

[48] Scott S, Knapp M, Henderson J, Maughan B (2001). Financial cost of social care exclusion: follow up study of antisocial children into adulthood. BMJ.

[49] Knapp M, McCrone P, Fombonne E, Beecham J, Wostear G (2002). The Maudsley long-term follow-up of child and adolescent depression: 3. Impact of comorbid conduct disorder on service use and costs in adulthood. Br J Psychiatry.

[50] Rivenbark JG, Odgers CL, Caspi A, Harrington H, Hogan S, Houts RM, Poulton R, Moffitt TE (2018). The high societal costs of childhood conduct problems: evidence from administrative records up to age 38 in a longitudinal birth cohort. J Child Psychol Psychiatry.

[51] Moffitt TE (1993). Adolescence-limited and life-course-persistent antisocial behavior: a developmental taxonomy. Psychol Rev.

[52] Kovess-Masfety V, Boyd A, van de Velde S (2014). Are there gender differences in service use for mental disorders across countries in the European Union? Results from the EU-World Mental Health survey. J Epidemiol Community Health.

[53] Buffel V, Van de Velde S, Bracke P (2014). Professional care seeking for mental health problems among women and men in Europe: the role of socioeconomic, family-related and mental health status factors in explaining gender differences. Soc Psychiatry Psychiatr Epidemiol.

[54] Vaidya V, Partha G, Karmakar M (2012). Gender differences in utilization of preventive care services in the United States. J Womens Health (Larchmt).

[55] Fleury M-J, Grenier G, Bamvita J-M, Perreault M, Caron J (2012). Determinants associated with the utilization of primary and specialized mental health services. Psychiatr Q.

[56] Evans-Lacko S, Knapp M (2018). Is manager support related to workplace productivity for people with depression: a secondary analysis of a cross-sectional survey from 15 countries. BMJ Open.

[57] Fergusson DM, Horwood LJ, Ridder EM (2005). Show me the child at seven: the consequences of conduct problems in childhood for psychosocial functioning in adulthood. J Child Psychol Psychiatry.

