# Supplementary material for: Effects of mental health status during adolescence on primary care costs in adulthood across three British cohorts
Source: Soc Psychiatry Psychiatr Epidemiol. 2023 Jun 26;59(6):917–28. doi: 10.1007/s00127-023-02507-y (PMC11116205; doi:10.1007/s00127-023-02507-y)
Supplement: Supplementary file 1 — Supplementary file1 (DOCX 28 kb) [file 127_2023_2507_MOESM1_ESM.docx]

**Supplementary Material for**

**Effects of mental health status during adolescence on primary care costs in adulthood across three British cohorts**

Accepted for publication in: **Social Psychiatry and Psychiatric Epidemiology**

Derek King,^1^ Petra C. Gronholm,^2^ Martin Knapp,^1^ Mauricio S. Hoffmann,^1,3,4^ Eva-Maria Bonin,^1^ Nicola Brimblecombe,^1^ Rajendra Kadel,^5^ Barbara Maughan,^6^ Nick O’Shea,^7^ Marcus Richards,^8^ Ties Hoomans,^1^ Sara Evans-Lacko ^1^

1 Care Policy and Evaluation Centre, London School of Economics and Political Science, London, UK

2 Health Service and Population Research Department, Institute of Psychiatry, Psychology & Neuroscience, King’s College London, London, UK

3 Department of Neuropsychiatry, Universidade Federal de Santa Maria, Avenida Roraima 1000, building 26, office 1446, Santa Maria, Brazil

4 Universidade Federal do Rio Grande do Sul, Rua Ramiro Barcelos 2350, Porto Alegre, Brazil

5 Public Health Wales, Policy and International Health Directorate, WHO CC on Investment for Health and Wellbeing, Cardiff, UK

6 Social, Genetic and Developmental Psychiatry Centre. Institute of Psychiatry, Psychology & Neuroscience, King’s College London, London UK

7 Chief Economist, Centre for Mental Health, London, UK

8 MRC Unit for Lifelong Health and Ageing at UCL, University College London, UK

Corresponding author: Derek King. E-mail: [d.king@lse.ac.uk](mailto:d.king@lse.ac.uk).

#### **Supplementary Table 1**

#### Descriptive information by cohort regarding data collection in key domains: adolescent mental health, GP visits

|  | **NSHD** | **NCDS** | **BCS70** |
| --- | --- | --- | --- |
| **Adolescent mental health** (ages at which problems were assessed) | | | |
| Conduct and emotional problems ^1^ | 13, 15 | 16 | 16 |
| Year and ages at which GP visits were assessed) ^2^ | | | |
| Year | 1972, 1977 and 1989 | 1991, 2000 and 2008 | 1996, 2000 and 2008 |
| Age | 26, 31 and 43 | 33, 42 and 50 | 26, 30 and 38 |

^1^ NSHD teacher report; NCDS & BCS parent report

^2^ Includes service contact described as: GP, GP following accident

**Supplementary Table 2**

National Survey of Health and Development – Distribution of demographic characteristics across values of adolescent mental health status indicators

|  | Conduct disorder | | | Emotional problems | | | Mental health problem severity | | | |
| --- | --- | --- | --- | --- | --- | --- | --- | --- | --- | --- |
|  | No  (%) | Yes  (%) | p-value | No  (%) | Yes  (%) | p-value | None  (%) | Mild/Moderate  (%) | Severe  (%) | p-value |
| Gender – female | 49.8 | 42.1 | 0.001 | 42.2 | 53.5 | 0.001 | 42.6 | 49.9 | 52.4 | 0.001 |
| Mother’s educ. – FT at age 16 + | 86.1 | 88.9 | 0.021 | 85.4 | 88.1 | 0.012 | 84.3 | 87.3 | 90.0 | 0.001 |
| Tenure – household home owned | 24.2 | 19.6 | 0.003 | 23.7 | 22.4 | 0.322 | 25.6 | 23.4 | 17.7 | 0.001 |
| Father’s social class |  |  | 0.001 |  |  | 0.007 |  |  |  | 0.001 |
| I | 6.4 | 4.5 |  | 6.8 | 5.1 |  | 7.4 | 5.7 | 3.9 |  |
| II | 20.3 | 15.7 |  | 20.6 | 17.7 |  | 22.1 | 19.2 | 13.7 |  |
| III | 48.67 | 49.3 |  | 48.2 | 49.5 |  | 48.0 | 48.5 | 51.1 |  |
| IV | 19.0 | 20.7 |  | 17.9 | 21.0 |  | 17.4 | 19.7 | 22.4 |  |
| V | 5.7 | 9.8 |  | 6.2 | 6.8 |  | 5.1 | 7.1 | 8.9 |  |
| Disability^1^ – Yes | 5.1 | 8.4 | 0.001 | 3.1 | 8.7 | 0.001 | 1.8 | 6.1 | 12.8 | 0.001 |
| Cognitive Ability ^2^ – standardised: mean (SD) | 103.7  (14.7) | 97.1  (16.2) |  | 103.3  (15.0) | 100.8  (15.5) |  | 105.4  (14.4) | 101.7  (14.9) | 96.8  (16.3) |  |

^1^ Teachers were asked about the study subject if “school work adversely affected by any physical disability at 13 years”, with a yes/no answer.

^2^ Cognitive ability using the National Foundation for Educational Research test.

**Supplementary Table 3**

National Child Development Study – Distribution of demographic characteristics across values of adolescent mental health status indicators

|  | Conduct disorder | | | Emotional problems | | | Mental health problem severity | | | |
| --- | --- | --- | --- | --- | --- | --- | --- | --- | --- | --- |
|  | No | Yes | p-value | No | Yes | p-value | None  (%) | Mild/Moderate  (%) | Severe  (%) | p-value |
| Gender – % female | 50.1 | 45.8 | 0.001 | 44.1 | 53.3 | 0.001 | 46.2 | 49.2 | 52.4 | 0.001 |
| Mother’s educ. – % FT at age 14/15 + | 79.7 | 88.2 | 0.001 | 80.9 | 83.7 | 0.001 | 78.5 | 83.8 | 86.0 | 0.001 |
| Tenure – % household home owned | 45.9 | 32.8 | 0.001 | 42.8 | 40.7 | 0.032 | 46.1 | 39.7 | 38.2 | 0.001 |
| Father’s social class (%) |  |  | 0.001 |  |  | 0.050 |  |  |  | 0.001 |
| I | 5.2 | 2.6 |  | 4.7 | 4.0 |  | 5.4 | 4.0 | 3.5 |  |
| II | 21.1 | 14.4 |  | 20.0 | 18.2 |  | 21.9 | 18.3 | 15.6 |  |
| III | 51.5 | 53.2 |  | 51.0 | 53.0 |  | 50.3 | 52.7 | 53.6 |  |
| IV | 14.0 | 16.6 |  | 14.7 | 15.0 |  | 14.2 | 14.9 | 15.8 |  |
| V | 7.6 | 12.3 |  | 8.8 | 9.3 |  | 7.6 | 9.3 | 11.0 |  |
| Disability^1^ – % Yes | 5.9 | 11.1 | 0.001 | 5.6 | 9.5 | 0.001 | 4.7 | 7.3 | 12.8 | 0.001 |
| General Ability ^2^ – standardised: mean (SD) | 102.4  (14.6) | 96.7  (14.4) |  | 101.5  (14.6) | 99.7  (14.8) |  | 99.9  (14.7) | 97.9  (14.6) | 98.5  (15.5) |  |

^1^ Parental interview form – NCDS 2008; Question 66: Taking into account the information you (interviewer) have obtained during the interview and any other relevant information, do you consider the child has any handicapping condition or disability?

^2^ General Ability Scale.

**Supplementary Table 4**

1970 British Cohort Study – Distribution of demographic characteristics across values of adolescent mental health status indicator

|  | Conduct disorder | | | Emotional problems | | | Mental health problem severity | | | |
| --- | --- | --- | --- | --- | --- | --- | --- | --- | --- | --- |
|  | No  (%) | Yes  (%) | p-value | No  (%) | Yes  (%) | p-value | None  (%) | Mild/Moderate  (%) | Severe  (%) | p-value |
| Gender – female | 52.3 | 45.6 | 0.001 | 48.2 | 54.3 | 0.001 | 50.0 | 51.1 | 53.0 | 0.112 |
| Mother’s educ. – FT at age 14/15 + | 99.1 | 98.1 | 0.001 | 99.1 | 98.7 | 0.118 | 99.1 | 99.0 | 98.4 | 0.080 |
| Tenure – household home owned | 68.3 | 53.4 | 0.001 | 67.6 | 63.3 | 0.001 | 69.8 | 64.0 | 59.2 | 0.001 |
| Father’s social class |  |  | 0.001 |  |  | 0.001 |  |  |  | 0.001 |
| I | 5.8 | 3.3 |  | 6.2 | 4.3 |  | 6.5 | 5.1 | 2.9 |  |
| II | 26.6 | 19.5 |  | 26.5 | 24.1 |  | 27.4 | 24.8 | 21.9 |  |
| III | 49.9 | 53.3 |  | 49.7 | 51.3 |  | 49.5 | 49.6 | 54.2 |  |
| IV | 12.3 | 14.5 |  | 12.1 | 13.6 |  | 11.6 | 13.6 | 13.6 |  |
| V | 5.5 | 9.4 |  | 5.5 | 6.7 |  | 4.9 | 7.0 | 7.4 |  |
| Disability^1^ – Yes | 0.9 | 2.1 | 0.001 | 0.7 | 1.8 | 0.001 | 0.7 | 0.9 | 2.8 | 0.001 |
| General Ability ^2^ – standardised: mean (SD) | 103.4  (14.2) | 96.9  (14.5) |  | 103.4  (14.2) | 100.8  (14.6) |  | 104.3  (14.1) | 101.4  (14.1) | 98.6  (15.2) |  |

^1^ Parental Interview Form – Question D7: Does your teenager have an impairment, a disability or a handicap? (By ‘impairment’ we mean a physical or mental abnormality/illness. By ‘disability’ we mean difficulty in doing one or more mental or physical activities that average 16 year olds can do. By ‘handicap’ we mean a disability which interferes with the opportunities that others take for granted, e.g. problems with access/facilities in public buildings; not being considered for jobs he or she could manage if given a chance; other people are put off without even knowing what he or she is like).

^2^ British Ability Scale.
